# Case report: *De novo* pathogenic variant in *WFS1* causes Wolfram-like syndrome debuting with congenital bilateral deafness

**DOI:** 10.3389/fgene.2022.998898

**Published:** 2022-10-18

**Authors:** Laura Alías, Miguel López de Heredia, Sabina Luna, Núria Clivillé, Lídia González-Quereda, Pía Gallano, Júlia de Juan, Albert Pujol, Santiago Diez, Susana Boronat, César Orús, Adriana Lasa, María del Prado Venegas

**Affiliations:** ^1^ Genetics Department, IIB Sant Pau, Hospital de la Santa Creu I Sant Pau, Barcelona, Spain; ^2^ U705—Centre for Biomedical Network Research on Rare Diseases (CIBERER), Instituto de Salud Carlos III, Madrid, Spain; ^3^ Centre for Biomedical Network Research on Rare Diseases (CIBERER), Instituto de Salud Carlos III, Madrid, Spain; ^4^ Ophthalmology Department, Hospital de la Santa Creu I Sant Pau, Barcelona, Spain; ^5^ Otorhinolaringologyst Department, Hospital de la Santa Creu I Sant Pau, Barcelona, Spain; ^6^ Otorhinolaringology Department, Hospital Esperit Sant, Santa Coloma de Gramenet, Spain; ^7^ Child Neurology Unit, Hospital de la Santa Creu I Sant Pau, Barcelona, Spain

**Keywords:** *WFS1* gene, congenital hearing loss, NGS, Wolfram-like syndrome, optic atrophy

## Abstract

**Background:** Congenital deafness could be the first manifestation of a syndrome such as in Usher, Pendred, and Wolfram syndromes. Therefore, a genetic study is crucial in this deficiency to significantly improve its diagnostic efficiency, to predict the prognosis, to select the most adequate treatment required, and to anticipate the development of other associated clinical manifestations.

**Case presentation:** We describe a young girl with bilateral congenital profound deafness, who initially received a single cochlear implant. The genetic study of her DNA using a custom-designed next-generation sequencing (NGS) panel detected a *de novo* pathogenic heterozygous variant in the *WFS1* gene related to Wolfram-like syndrome, which is characterized by the presence of other symptoms such as optic atrophy. Due to this diagnosis, a second implant was placed after the optic atrophy onset. The speech audiometric results obtained with both implants indicate that this work successfully allows the patient to develop normal speech. Deterioration of the auditory nerves has not been observed.

**Conclusion:** The next-generation sequencing technique allows a precise molecular diagnosis of diseases with high genetic heterogeneity, such as hereditary deafness, while this was the only symptom presented by the patient at the time of analysis. The NGS panel, in which genes responsible for both syndromic and non-syndromic hereditary deafness were included, was essential to reach the diagnosis in such a young patient. Early detection of the pathogenic variant in the *WFS1* gene allowed us to anticipate the natural evolution of the disease and offer the most appropriate management to the patient.

## Introduction

Congenital deafness may be caused by genetic etiology in more than 60% of cases in developed countries. These genetic cases could either be syndromic (SHL, 30%), in which the hearing loss is accompanied by other associated clinical manifestations, or non-syndromic (NSHL, 70%), in which the hearing loss remains as the only symptom in the patient.

More than 400 syndromes manifest SHL, and among them, Usher, Pendred, and Wolfram syndromes have been described. Identifying patients who may develop SHL when the only manifestation presented by the patient is hearing loss might be highly valuable for proper patient management.

Wolfram syndrome (WS; OMIM #222300; previously known as DIDMOAD) is a rare (1 in 500,000–1,000,000) (Blanco-Aguirre et al., 2015) autosomal recessive disease, initially described as a combination of early-onset diabetes mellitus, progressive optic nerve atrophy, diabetes insipidus, and sensorineural hearing loss associated with other variable clinical manifestations (Barrett et al., 1995).

There are two types of WS with many overlapping features, type I and type II, differentiated by their genetic cause due to pathogenic variants in the wolframin ER transmembrane glycoprotein (*WFS1*), most frequently, and *CISD2* genes, respectively. *WFS1* pathogenic variants have been linked to Wolfram and Wolfram-like syndromes, which include cases with just one pathogenic variant in heterozygosis not meeting the WS diagnostic criteria (Barrett et al., 1995; Inoue et al., 1998; Takei et al., 2006; Heredia et al., 2013; Blanco-Aguirre et al., 2015). Wolfram syndrome is an autosomal recessive disorder caused by bi-allelic variants in *WFS1*, whereas Wolfram-like syndrome is a dominant condition caused by a single heterozygous pathogenic variant in *WFS1*. This gene encodes for an endoglycosidase H-sensitive protein called wolframin (Inoue et al., 1998) localized to the endoplasmic reticulum (ER) membrane and secretory granules. Wolframin plays a role in Ca^2+^ homeostasis regulation at the cellular and ER levels, which contributes to the quality control systems for protein folding and regulation of the ER stress response. Wolframin is highly expressed in the heart, lungs, specific regions of the brain, pancreas, liver, kidney, skeletal muscle, optical nerve, and the auditory pathway. There are more than 100 reported pathogenic genetic variants that cause WS due to the abnormal wolframin activity or protein levels (Heredia et al., 2013); however, genotype–phenotype correlations have not been described.

Bi-allelic loss of function variants in wolframin trigger a cascade of ER and mitochondrial dysfunction that ultimately leads to apoptosis and cellular death (Takei et al., 2006; Bonnet Wersinger et al., 2014), especially of pancreatic beta-cells and neurons. This effect on neurons leads to neurodegeneration due to an evident cellular degradation of myelin (Lugar et al., 2016).

Molecular genetic studies have shown that wolframin deficiency may impair early neuronal survival and delay neuronal development (Cagalinec et al., 2016). Furthermore, postmortem histopathological studies specify that the most affected brain regions in WS are the sensory pathways, brainstem, cerebellum, and hypothalamus. In the visual system, the optic nerves appear grossly atrophic, and microscopic examination reveals the loss of retinal ganglion neurons and myelinated axons throughout the visual pathways with relative preservation of the visual cortex. Within the auditory pathway, studies have found loss of the organ of Corti (the functional unit of the inner ear) in the basal turns of the cochlea, fibers in the cochlear nerve, and neurons in the cochlear nuclei and inferior colliculus (Shannon et al., 1999). In addition, altered wolframin disturbs the balance of calcium ions in the inner ear, which interferes with the hearing process (Takeda et al., 2001).

In this work, we describe a girl, who is clinically diagnosed with congenital deafness, in which NGS results determined the presence of a heterozygous *de novo* missense variant in *WFS1* and was predicted to be pathogenic by different *in silico* prediction algorithms.

We describe the genotype–phenotype relationship in this patient and the early treatment performed, emphasizing the importance of the exhaustive and personalized follow-up that allowed us to anticipate the onset of other clinical symptoms related to WS, thus improving patient’s quality of life through an early management.

## Case presentation

**FIGURE 1:**
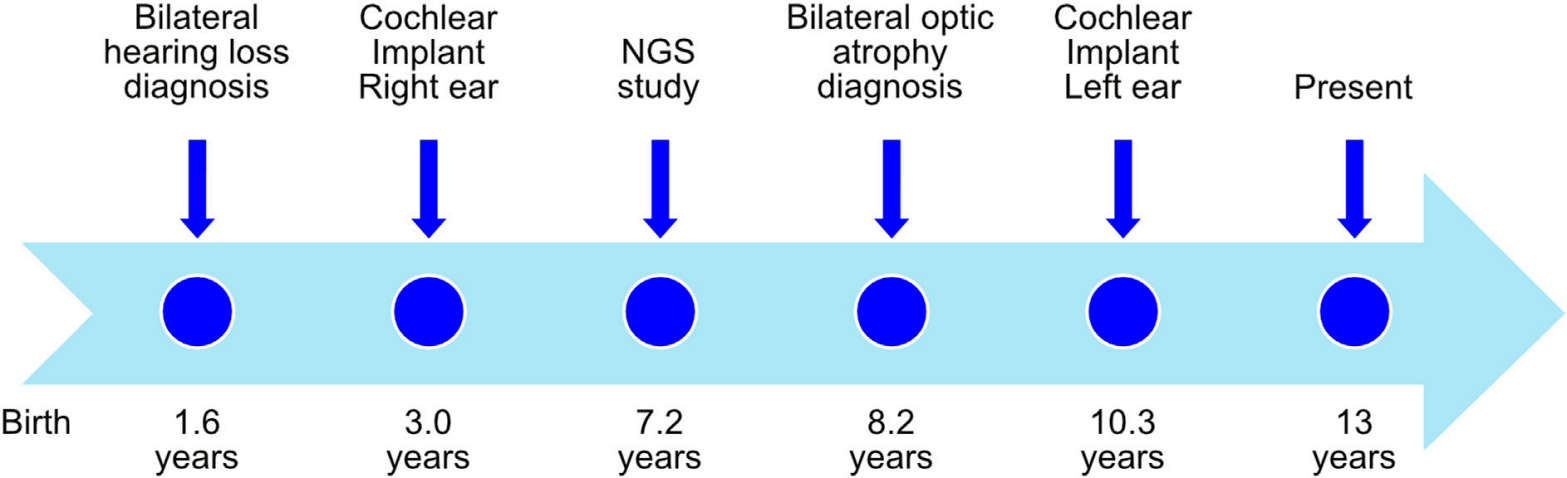
Timeline with relevant episodes in the case report presented.

**FIGURE 2:**
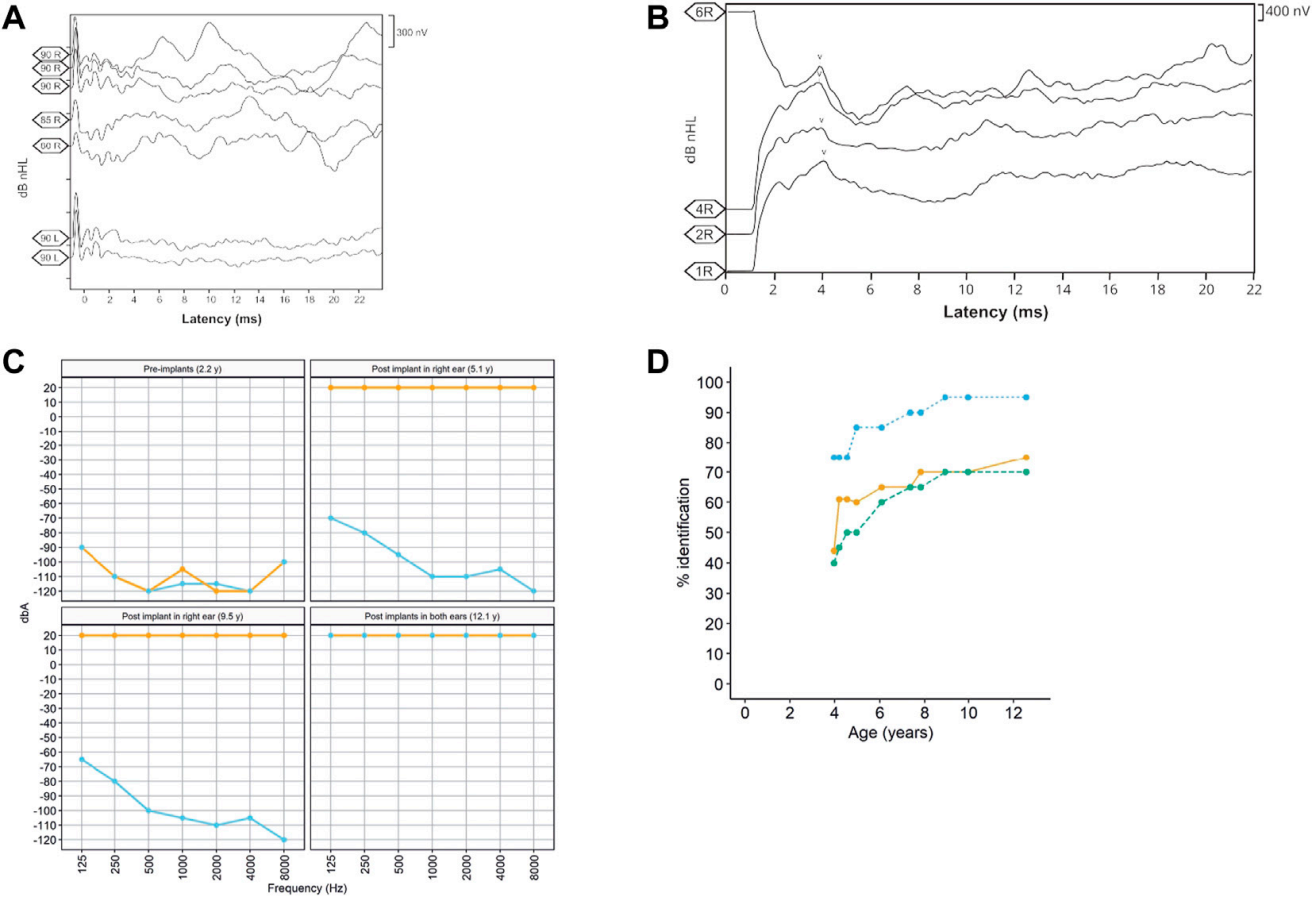
**(A)** Auditory brainstem potential performed at an age of 1 year, showing the potentials performed in both the right (R) and left (L) ears at different dB HL (90, 85, and 80) levels. **(B)** Auditory brainstem potential performed at an age of 13 years using the implanted electrode in the right ear. The V-wave curve (v) recorded from the electrodes 1, 2, 4, and 6 of the cochlear implant in the right ear (R). **(C)**. Pre- and post-implant audiometries showing the age when performed. Orange = right ear and sky blue = left ear. **(D)**. Evolution of the auditory and speech perception tests over patient age. The panel shows the percentage of word discrimination at 60 dB (bluish green-dashed line and dots), the percentage of consonant identification (the orange solid line and dots), and the percentage of vowel identification at 60 dB (the sky blue dotted line and dots) at tested ages.

The patient, a 13-year-old female (Figure 1), without previous familial history of hearing loss, at an age of 1.6 years was diagnosed with profound bilateral deafness in both ears by means of otoacoustic emissions, brainstem-evoked potentials, and audiometry. Otoacoustic emissions showed no response in both ears, and no V-wave at 90 dB nHL was found when performing brainstem-evoked potentials (Figure 2A), and also, no response was obtained in pure-tone audiometry when stimulated at high intensity at all frequencies studied (Figure 2C). Since a hearing response was not obtained with the most powerful hearing aids on the market, a cochlear implant was chosen.

The radiological study performed to evaluate cochlear implant placement with cranial computed tomography (CT) and magnetic resonance imaging (MRI) showed neither disease-related changes in the central nervous system nor cochlear, nerve, or auditory pathway malformations. A cochlear implant (CI 513, cochlear) was placed in the right ear when the patient was 3 years old.

In order to see if there is a genetic cause for patient’s deafness, an NGS genetic study was performed when the patient was 7.2 years old, using an in-house TruSeq Custom Amplicon panel (TSCA) from Illumina (Supplementary Table S1). Amplicons were then paired-end sequenced using a MiSeq sequencer (Illumina), with a read length of 150 base pairs. Different variants were detected (Table 1 and Supplementary Figure S1) by VariantStudio and DNAnexus software applications, and only one of them was *in silico* predicted as pathogenic. A c.2051C>T nucleotide change was observed in the g.6303573 position of chromosome 4 (GRCh37), causing an alanine exchange for valine (p.Ala684Val) in *WFS1* gene (NM_006005.3). Sanger sequencing confirmed the presence of a heterozygous c.2051C>T variant in the patient’s *WFS1* gene but not in either of her parents (Supplementary Figure S1). Therefore, the pathogenic variant in our patient was categorized as *de novo*.

**TABLE 1:** Genetic variants detected in the affected patient after the NGS analysis. Six of them were identified as synonymous variants or classified as benign/likely benign by *in silico* software. The variants in bold were selected because its genetic consequence according to the ACMG guidelines (Richards et al., 2015) and being predicted as pathogenic by several *in silico* software applications (ClinVar, PolyPhen, and SIFT). GRCh37 (hg 19) was considered as the reference. The RNA splicing effect in missense and synonymous variants was discarded by Alamut software.

Gene	Chr	Coordinate	Freq	Read depth	Depth variant	HGVSc	HGVSp	Consequence	ClinVar	PolyPhen	SIFT	dbSNP ID	ACMG criteria	Classification
*ADGRV1*	5	89,990,447	48,2	110	53	NM_032119.3: c.7874G>A	p.Arg2625His	Missense	Likely benign	Benign (0.018)		rs201214794	BA1, BS1, and BS2	Class 1—benign
*ADGRV1*	5	90,020,923	46,3	164	76	NM_032119.3: c.9927T>G	p.Pro3309Pro	Synonymous				rs16869042	BA1 and BS2	Class 1—benign
*CDH23*	10	73,569,731	31	84	26	NM_022124.5: c.8877C>T	p.Ile2959Ile	Synonymous				rs373709237	PM2, BP7, and BP6	Class 2—likely benign
*GJA1*	6	121,768,897	30,9	139	43	NM_000165.3: c.904A>G	p.Asn302Asp	Missense		Benign (0.012)	Tolerated (0.16)	rs775532447	PM2 and PP2	Class 3—VUS
***GJA1***	6	**121,768,924**	**30,9**	139	43	**NM_000165.3: c.932delC**	**p.Ala311ValfsTer37**	**Frameshift**				**rs778110855**	**PVS1**	**Class 4**—**likely pathogenic**
*GJA1*	6	121,769,050	30,5	141	43	NM_000165.3: c.1057T>C	p.Leu353Leu	Synonymous					PM2, BP4, and BP7	Class 2—likely benign
*MY O 7A*	11	76,883,797	25	12	3	NM_000260.3: c.1801G>A	p.Ala601Thr	Missense		Benign (0.245)	Tolerated (0.34)	rs782481491	PM2	Class 3—VUS
***WFS1***	4	**6,303,573**	**49,1**	322	158	**NM_006005.3: c.2051C>T**	**p.Ala684Val**	**Missense**	**Pathogenic**	**Probably damaging (0.992)**	**Deleterious (0)**	**rs387906930**	**PM2, PM5, PM1, PP3,** and **PP5**	**Class 5—pathogenic**

Chr and Coordinate, are the chromosome and coordinates where the gene is located. Freq indicates the variant frequency detected in sequencing. HGVSc and HGVSp shows the naming at DNA and protein levels according to HGVS convenctions.

Additionally, NGS analysis showed a heterozygous deletion c.932delC in the g.121768924 position of chromosome 6 (GRCh37), resulting in p.Ala311Valfs37* in the *GJA1* gene (NM_00165.3). After Sanger sequencing validation, an exhaustive analysis with BLAST® (NCBI) showed that the sequence corresponded instead to the FER tyrosine kinase (*FER*) pseudogene in chromosome 5 (NG_011445.2) (Supplementary Figure S1), which presents more than 90% homology with the *GJA1*’s sequence.

We ruled out the presence of the p.Arg445His missense pathogenic variant in the *OPA1* gene in all family members by Sanger sequencing as *OPA1* pathogenic variants are the most frequent genetic cause behind optic atrophy associated with deafness (Amati-Bonneau et al., 2003).

Given the result of the genetic study and to anticipate the possible appearance of other associated symptoms, the patient was referred to an endocrinologist, who has, so far, ruled out the presence of diabetes mellitus or insipidus, an ophthalmologist, and also a neuropediatrician.

**FIGURE 3:**
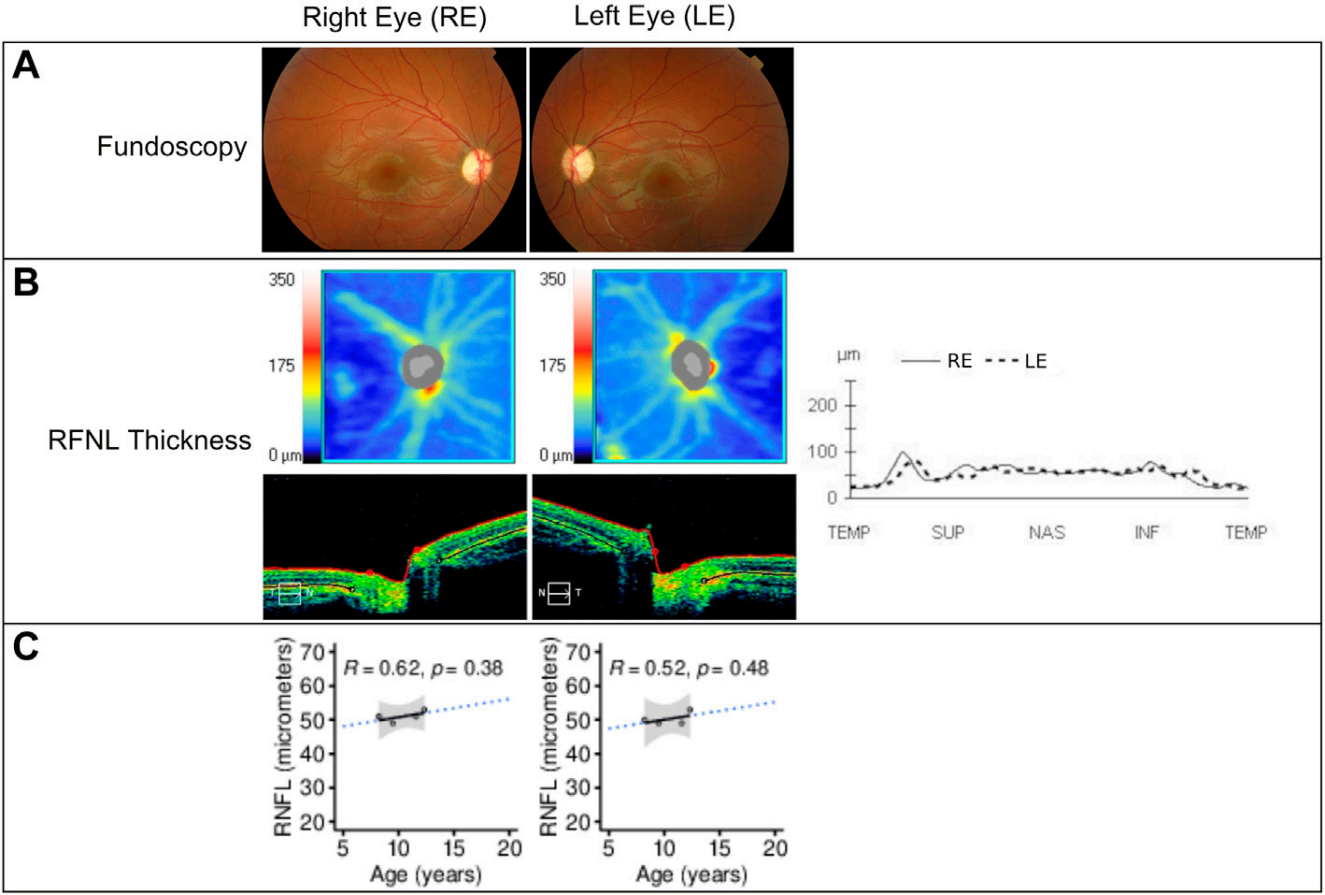
**(A)** Fundoscopy showing optic nerve pallor and bilateral atrophy. **(B)** Retinal nerve fiber layer (RNFL) analysis by time-domain optical coherence tomography of both eyes at 12.3 years of age using Cirrus HD-OCT and SW Ver 11.5.254532 (Carl Zeiss Meditec), showing from the top to bottom the RNFL thickness maps and the nasal (N) to temporal (T) quadrants’ extracted tomographs. Graphical representation of the RNFL thickness from all quadrants is also shown at the right of the images. **(C)** RNFL thickness (in micrometers) in function of the patient’s age showing linear regression (black solid line), 95% confidence interval (gray area), and prediction outside measured ages (blue dotted line). The Pearson correlation coefficient and *p*-value are shown in each panel. RE = right eye; LE = left eye; µm = micrometers.

The patient’s initial eye examination performed at the age of 8.2 years included visual acuity (VA), color vision test, biomicroscopy, funduscopy, visual field, optical coherence tomography (OCT), and visual-evoked potentials (Figure 3). The ophthalmologic manifestation in the patient included reducing acuity, dyschromatopsia, and visual field deficit. Despite showing correct VA at log MAR 0.0 in each eye and no alterations in the color test, fundoscopy and retinography showed bilateral papillary pallor and marked thinning of the peripapillary RNFL retinal nerve fiber layer on OCT. No signs of vascular narrowing and/or diabetic retinopathy were detected, all suggestive of optic atrophy. In successive ophthalmologic controls, these findings have remained stable without progression. This pathology is not subject to optical correction.

In the context of the Spanish public health system, when this patient was diagnosed with deafness, only one cochlear implant was approved by the authorities. However, because the patient had both optic atrophy and profound bilateral deafness, a second cochlear implant was allowed to be placed in the left ear at the age of 10.3 years. After 9 years with the cochlear implant in the right ear and then 3 years in the left ear, we studied the evolution of the speech recognition tests and the post-implant neural response. Auditory perception and speech tests improved significantly after the placement of both cochlear implants (Figure 2D). The hearing threshold in pure-tone audiometry with both cochlear implants at conversational frequencies was 20 dB. Speech audiometry results varied in both ears due to the time of hearing deprivation and years of follow-up after placement of both implants. After 9 years of follow-up since the first cochlear implant, the results in the right ear showed 70% speech recognition at a sound loudness level (SPL) of 70 dB, while the left ear results showed 65% speech recognition at the same intensity. V-wave curves were recorded at electrodes 6, 11, 16, and 22 at a level between 140 and 167 CL, showing good morphology and replicability, which suggests good neural conduction from the auditory nerve to the anatomical generator (the inferior colliculus) of the brainstem (Figure 2B).

## Discussion

We describe a patient diagnosed with congenital deafness, who presents a heterozygous *de novo* missense pathogenic variant (p.Ala684Val) in the *WFS1 gene*. The p.Ala684Val variant 1) is not observed in the gnomAD v2.1.1 dataset, 2) has been previously reported on 16 independent occasions as pathogenic/likely pathogenic with strong evidence (ClinVar ID: VCV000030556), and 3) exhibited a different missense change at the same codon (p.Ala684Thr) which has been reported to be associated with WS in compound heterozygosis (Waschbisch et al., 2011). Therefore, this variant could be classified as pathogenic, according to the recommendation of ACMG/AMP guidelines (Richards et al., 2015). This genetic variant was previously reported in compound heterozygosity in a patient with full WS (Tessa et al., 2001) and in six families in heterozygosis linked to autosomal-dominant WS-like syndrome (Rendtorff et al., 2011), showing a wide spectrum of the severity and type of clinical manifestations. Different pathogenic variants in *WFS1* may give rise to different disease phenotypes, but genotype–phenotype correlations for either WS or Wolfram-like syndrome have been elusive, especially due to the low number of described patients and the large number of reported variants (Heredia et al., 2013).

As the patient has not developed diabetes mellitus and only one mutated allele was found in *WFS1*, the patient was diagnosed with Wolfram-like syndrome, according to the available guidelines (Tranebjærg et al., 2020).

Expression of the p.Ala684Val pathogenic variant in HEK cells showed a significantly decreased protein expression compared to wild-type wolframin (Rendtorff et al., 2011). As the variant has been shown in heterozygosis, we could deduce from these data that the patient might have reduced wolframin levels.

The clinical manifestations of the patient described in this work are related to sensory neurodevelopmental disorders (congenital bilateral deafness and progressive optic atrophy), suggesting that the neurons of the sensory organs (retinal ganglion cells, hair cells, and auditory nerves) would be more susceptible to the wolframin insufficiency caused by the *WFS1* genetic variant present in this patient. It was believed that the neurological manifestations appeared in the late stages of the disease, but recent evidences gathered from WS patients indicate that some of these neurological abnormalities are present even at an early age, as seen in the present case (Hershey et al., 2012).

Some neuropathological studies have been performed based on cranial MRI and postmortem brain histopathological studies of patients affected by WS. Through these studies, it has been possible to identify the brain regions and structures involved and affected by WS. Interestingly, the most affected brain regions are the sensory pathways, brainstem, cerebellum, and hypothalamus (Shannon et al., 1999).

In the visual system of WS patients, the optic nerves appear grossly atrophic, and microscopic examination reveals loss of retinal ganglion neurons and myelinated axons in all visual pathways, with relative preservation of the visual cortex (Lugar et al., 2016). Grenier et al. (2016) described a progressive decrease in visual acuity (VA) and significantly decreased retinal nerve fiber layer (RNFL) thickness in a group of patients affected by WS compared to a group of patients with Wolfram-like syndrome. We observed a decrease to 53 µm of the RNFL in the right eye and 52 µm in the left eye, respectively, during the 3-year follow-up of our patient’s optic atrophy. This result is similar to those described by Grenier. However, VA and visual fields were not affected. This could be explained by the young age of the patient and the short follow-up time.

Within the auditory pathway, published studies have found that pathogenic variants in the *WFS1* gene affect the entire auditory pathway, from the organ of Corti to the nuclei of the pons (Genís et al., 1997). Likewise, molecular genetic studies have shown that wolframin deficiency can impair early neuronal survival and delay neuronal development (Cagalinec et al., 2016). *WFS1* is expressed during brain development, and molecular pathways affected by wolframin deficiency also play crucial roles in early brain development, for example, neurogenesis, neuronal migration, or myelination. Recent neuroimaging studies suggest that abnormal myelin development is a primary neuropathologic feature of WS that is observed from an early age (Lugar et al., 2019). One possible explanation is that wolframin deficiency impairs the function of myelinating oligodendrocytes and interferes with myelin development. Another explanation could be that ER stress triggers oligodendrocyte death and facilitates myelin degeneration. However, functional studies of the *WFS1* gene in oligodendrocytes and glial cells are limited (Samara et al., 2019).

The damage caused by the *WFS1* genetic variant described in the studied patient at the level of the auditory pathway and the brain has not been related. The correlation of specific variants with the age at which the different symptoms in WS or Wolfram-like syndromes debut or progress has not been related either. It is believed that based on the few patients with the p.Ala684Val variant described, including the patient described here it could first affect the auditory and optical pathways and that the variability in onset ages might be due to the activity of undescribed modulating genes. Interestingly, another pathogenic variant affecting the same amino acid (p.Ala 684Thr) has been described in WS patients (Waschbisch et al., 2011). The patient showed the full characteristic spectrum of WS symptoms, probably because two compound heterozygotic pathogenic variants were found in *WFS1*. Current data available suggest that the spectrum of symptoms shown by WS and Wolfram-like patients might be due to wolframin levels in the pancreatic beta-cells and visual and auditory pathways, but still, the difference in wolframin levels does not explain the variability in the onset and order of appearance of the symptoms.

There is currently no effective, scientifically proven treatment to intervene or remediate damage to the visual or auditory pathways in WS or Wolfram-like syndrome. However, there is a technological management that helps restore deafness. The cochlear implant replaces the function of the cochlea and would explain the good results obtained in the patient if wolframin was located only at the level of the cochlea. However, as previously mentioned, the presence of wolframin is distributed along the auditory pathway, which raises the question if the auditory nerve atrophy shown in WS patients might be due to the partial or complete depletion of wolframin or by the lack of stimuli. The evidence from this patient is that the auditory nerve atrophy is secondary, so an early intervention might conserve its functionality. There is no evidence in the literature on how auditory nerve stimulation through the cochlear implant would act favoring the transmission of sound, connecting the patient to the “sound world” and allowing the comprehension of words. We hypothesize that the cochlear implant stimulates the auditory nerve, resynchronizing the ascending auditory pathway, presumably delaying the process of axonal demyelination and allowing the patient to perceive and understand sounds. All this would be supported by the fact that we have observed the good results obtained from the auditory point of view in the patient with the placement of both cochlear implants at an early age, before demyelination progressed to the axonal level. These results have been demonstrated at the audiometric and speech recognition levels and by obtaining a correct neuronal response when stimulating the auditory nerves through a cochlear implant.

Although deep phenotypic descriptions of patients with WS and Wolfram-like syndrome are still needed to deepen our knowledge on the evolution of the syndromes, patients with deleterious pathogenic variants in *WFS1* would significantly benefit from early cochlear implantation to preserve a fully functional auditory pathway, thus improving their quality of life.

## Conclusion

NGS’s usefulness allowed us to detect the presence of pathogenic variants in genes related to WS in a patient with congenital deafness without a family history. We would like to highlight the importance of including the study of the genes responsible for hereditary syndromic hearing loss in patients, who are only affected by this disease without a family history, especially when these patients are very young.

An early detection of the pathogenic variant in the *WFS1* gene has been essential to anticipate the natural evolution of the clinical manifestations and to successfully treat the patient described in this work. As a result of early intervention with cochlear implants, the functionality of the auditory pathway might be maintained, which was critical for the patient’s quality of life after the onset of optic atrophy.

Currently, the patient is under the care of an otorhinolaryngologist, endocrinologist, ophthalmologist, and audiologist, as well as a psychologist, because mood disorders have been related to *WFS1*-associated diseases.

A complete genomic analysis, such as exome or whole-genome sequencing, should be considered in the near future in cases of patients presenting with congenital deafness, especially when hearing loss gene panel studies are inconclusive, in order to be able to identify genetic variants not described in the literature, particularly in those cases of SHL where the appearance of other clinical manifestations can be anticipated.

## Data Availability

The datasets for this article are not publicly available due to concerns regarding participant/patient anonymity. Request to access the datasets should be directed to the corresponding author.
