# Supplementary material for: Case report: De novo pathogenic variant in WFS1 causes Wolfram-like syndrome debuting with congenital bilateral deafness
Source: Front Genet. 2022 Oct 18;13:998898. doi: 10.3389/fgene.2022.998898 (PMC9623256; doi:10.3389/fgene.2022.998898)
Supplement: Supplementary file 1 [file Table1.DOCX]

**Supplementary information for:**

**Case Report: De novo pathogenic variant in *WFS1* causes Wolfram-like syndrome debuting with congenital bilateral deafness.**

Laura Alías^1,2,^*, Miguel López de Heredia^3^, Sabina Luna^4^, Núria Clivillé^1^, Lídia González-Quereda^1,2^, Pía Gallano^1,2^, Júlia de Juan^5^, Albert Pujol^5^, Santiago Diez^6^, Susana Boronat^7^, César Orús^5^, Adriana Lasa^1,2^, María del Prado Venegas^5^.

^1^Genetics Department, IIB Sant Pau, Hospital de la Santa Creu i Sant Pau, Barcelona, Spain.

^2^U705 - Centre for Biomedical Network Research on Rare Diseases (CIBERER), Instituto de Salud Carlos III, Madrid, Spain.

^3^Centre for Biomedical Network Research on Rare Diseases (CIBERER), Instituto de Salud Carlos III, Madrid, Spain.

^4^Ophthalmology Department, Hospital de la Santa Creu i Sant Pau, Barcelona, Spain. ^5^Otorhinolaringologyst Department. Hospital de la Santa Creu i Sant Pau, Barcelona, Spain.

^6^Otorhinolaringology Department, Hospital Esperit Sant, Sta. Coloma de Gramenet, Spain.

^7^Child Neurology Unit, Hospital Sant Pau, Barcelona, Spain.

# Supplementary methods

# Genetic studies

Considering that hereditary hearing loss (HHL) shows a deep genetic heterogeneity and that the patient did not present any other associated pathology, the NGS tool was selected. The in-house custom panel contained the most prevalent deafness related genes described in the literature at that time (HSantPau_HPpanelv.01, available under request).

Genomic DNA was automatically extracted from peripheral leukocytes using the salting out procedure (Autopure, Qiagen) after written informed consent was obtained from the index case and other family members.

To detect genetic variants involved in hearing loss we designed a TruSeq custom amplicon panel (TSCA) from Illumina© (Supp. Table 1). Amplified samples following the TSCA protocol were loaded onto the MiSeq instrument according to manufacturer’s instructions (Illumina©). Amplicons were then pair-end sequenced in a MiSeq sequencer (Illumina) with a read-length of 150bp. VariantStudio software® v3.0 (Illumina©) and DNAnexus® software (DNAnexus©) were used to analyze the results. Our design let us cover the 98 % of the coding and exon-intron boundaries (+/- 20 pb from the exons) of the genes included into the panel.

Sanger sequencing was performed to: 1) validate NGS results 2) discard genomic variants located in the not covered coding regions of the TSCA panel. 3) screen for the presence of the p.Arg445His missense mutation in the *OPA1* gene following the PCR conditions described by Amati-Bonneau *et al*. (1). All the primer sequences, annealing temperatures and PCR conditions were adapted from the literature and available upon request.

Sanger sequencing reaction was performed with Big Dye v1.1 Terminator Reaction Kit on an ABI Prism ® 3500 Dx Capillary DNA Sequencer unit according to manufacturer’s protocol (Life Technologies Corporation).

All mutations and genetic variants were numbered according to the first translated base of the sequenced genes and variant sequences were designated according to standard nomenclature guidelines (2).

To investigate the functional impact of the identified genetic variants, we used the ALAMUT® VISUAL PLUS 1.2 software. This software uses the following relevant prediction tools: i) Splice Site Finder-like, MaxEntScan, NNSPLICE, GeneSplicer, Human Splicing Finder and ESE for splicing prediction ii) Align GVGD, SIFT, MutationTaster, PolyPhen-2 and KD4v for missense pathogenicity prediction.

# Post implant neural study

Auditory brainstem potentials were performed through the cochlear implant (CI 513, Cochlear) in the right ear to verify the auditory pathway response. Stimulation was performed on eABR at electrodes 21, 16, 11 and 6. Interacoustics Eclipse EP 25 with Cochlear POD interface and Custom Sound 6.3 software were used. Impedances of the electrodes placed on the Vertex, left mastoid and zygomatic (less than 3 kOhm) were verified. The criterion for threshold determination was by visual inspection by performing stimulation starting at 100 CL and increasing the level of stimulation by 10.

A total of 18 stimulations were performed on the eABR with the following parameters: Current levels between 100 and 168 (CL), stimulation modes MP1+2 pulse width of 100 µS, stimulation speed of 35 Hz and up to 1024 repetitions, standard and reverse were used. The acquisition parameters were acquisition and presentation window of 23 mS, amplification of 40 µV, filters of 33-2500Hz with deactivated net filter at 50 Hz and up to 2000 repetitions.

# Supplementary tables

**Supplementary table 1.** Hearing loss related genes sequenced by our custom-TSCA panel. Genes that are responsible for causing deafness more frequently in the Spanish population are highlighted in bold letters.

| Non Syndromic (70%) | | Syndromic (30%) | |
| --- | --- | --- | --- |
| Connexins | **GJB2 (Cx26)** | USH1 | MYO7A |
|  | GJB6 (Cx30) |  | USH1C |
|  | GJB3 (Cx31) |  | CDH23 |
|  | GJA1 (Cx43) |  | PCDH15 |
| OTOF | |  | USH1G |
| COCH | | USH2 | **USH2A** |
| WFS1 | |  | GPR98 |
| POU3F4 | |  | DFBN31 |
| Mitochondrial | **MT-RNR1** | USH3 | CLRN1 |
|  | MT-TS1 | Pendred | **SLC26A4** |
|  | MT-TL1 |  | FOXI1 |

# Supplementary figures

**Supplementary figure 1.** Genetic variants in proband. **A.** Sanger chromograms showing the bases surrounding the mutated position in *WFS1* in the proband and progenitors **B.** Sanger chromograms showing the bases surrounding the mutated position in *FER* in the proband. **C.** Alignment of *FER* and *GJA1* gene sequences. The nucleotides marked in yellow show the differences between both sequences. The mutated nucleotide in the proband is labeled in red.


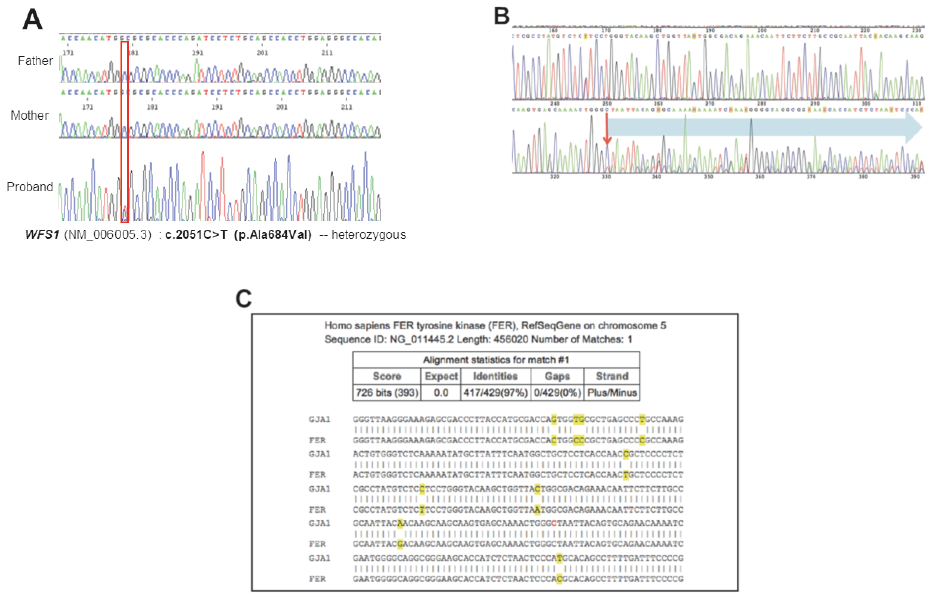


# Supplementary references

1. Amati-Bonneau P, Odent S, Derrien C, Pasquier L, Malthiéry Y, Reynier P, Bonneau D. The association of autosomal dominant optic atrophy and moderate deafness may be due to the R445H mutation in the OPA1 gene. *Am J Ophthalmol* (2003) **136**:1170–1171. doi: 10.1016/S0002-9394(03)00665-2

2. den Dunnen JT, Antonarakis SE. Mutation nomenclature extensions and suggestions to describe complex mutations: a discussion. *Hum Mutat* (2000) **15**:7–12. doi: 10.1002/(SICI)1098-1004(200001)15:1<7::AID-HUMU4>3.0.CO;2-N
